# Which surrogate insulin resistance indices best predict coronary artery disease? A machine learning approach

**DOI:** 10.1186/s12933-024-02306-y

**Published:** 2024-06-21

**Authors:** Seyed Reza Mirjalili, Sepideh Soltani, Zahra Heidari Meybodi, Pedro Marques-Vidal, Danial Dehghani Firouzabadi, Reza Eshraghi, David Restrepo, Hamed Ghoshouni, Mohammadtaghi Sarebanhassanabadi

**Affiliations:** 1 Yazd Cardiovascular Research Center, Non-Communicable Diseases Research Institute, Shahid Sadoughi University of Medical Sciences, Yazd, Iran; 2 Department of Medicine, University of California San Francisco, San Francisco, USA; 3 Department of Internal Medicine, BH10-642, Rue du Bugnon 46, Rue du Bugnon 46, Lausanne, CH-1011 Switzerland; 4 Student Research Committee, Shahid Sadoughi University of Medical Sciences, Yazd, Iran; 5 Student Research Committee, Kashan University of Medical Sciences, Kashan, Iran; 6 Laboratory for Computational Physiology, Massachusetts Institute of Technology, Cambridge, MA USA; 7 Telematics Department, University of Cauca, Popayán, Cauca, Colombia; 8 Rajaie Cardiovascular Medical and Research Center, Iran University of Medical Sciences, Tehran, Iran

**Keywords:** Cardiovascular diseases, Metabolic diseases, Public Health, Machine learning

## Abstract

**Background:**

Various surrogate markers of insulin resistance have been developed, capable of predicting coronary artery disease (CAD) without the need to detect serum insulin. For accurate prediction, they depend only on glucose and lipid profiles, as well as anthropometric features. However, there is still no agreement on the most suitable one for predicting CAD.

**Methods:**

We followed a cohort of 2,000 individuals, ranging in age from 20 to 74, for a duration of 9.9 years. We utilized multivariate Cox proportional hazard models to investigate the association between TyG-index, TyG-BMI, TyG-WC, TG/HDL, plus METS-IR and the occurrence of CAD. The receiver operating curve (ROC) was employed to compare the predictive efficacy of these indices and their corresponding cutoff values for predicting CAD. We also used three distinct embedded feature selection methods: LASSO, Random Forest feature selection, and the Boruta algorithm, to evaluate and compare surrogate markers of insulin resistance in predicting CAD. In addition, we utilized the ceteris paribus profile on the Random Forest model to illustrate how the model’s predictive performance is affected by variations in individual surrogate markers, while keeping all other factors consistent in a diagram.

**Results:**

The TyG-index was the only surrogate marker of insulin resistance that demonstrated an association with CAD in fully adjusted model (HR: 2.54, CI: 1.34–4.81). The association was more prominent in females. Moreover, it demonstrated the highest area under the ROC curve (0.67 [0.63–0.7]) in comparison to other surrogate indices for insulin resistance. All feature selection approaches concur that the TyG-index is the most reliable surrogate insulin resistance marker for predicting CAD. Based on the Ceteris paribus profile of Random Forest the predictive ability of the TyG-index increased steadily after 9 with a positive slope, without any decline or leveling off.

**Conclusion:**

Due to the simplicity of assessing the TyG-index with routine biochemical assays and given that the TyG-index was the most effective surrogate insulin resistance index for predicting CAD based on our results, it seems suitable for inclusion in future CAD prevention strategies.

## Introduction

Globally, cardiovascular diseases (CVDs) continue to significantly impact mortality rates and overall health outcomes [1]. Coronary artery disease (CAD) stands out as the most prevalent type among cardiovascular diseases (CVDs), exhibiting noticeable increases in its prevalence and incidence across the majority of countries [2]. From 1990 to 2019, the number of deaths and disability-adjusted life years (DALYs) caused by CAD has risen steadily. In 1990, there were around 5 million deaths and 120 million DALYs, but in 2019, there were 9.14 million deaths and 182 million DALYs [2]. This emphasizes the urgent need for precise identification of risk factors to predict and prevent CAD.

Insulin resistance is commonly regarded as one of the key risk factors for predicting CAD [3–5]. It is associated with chronic low-grade inflammation [6] which can lead to pro-coagulation states [7], decreased bioavailability of nitric oxide, and subsequently impaired endothelial function [8]. Further, insulin resistance can activate the sympathetic nervous system and reduce vagal activity, resulting in the activation of the renin-angiotensin-aldosterone system and kidney sodium retention, ultimately causing higher blood pressure and cardiovascular damage [9]. Remarkably, despite its considerable importance, it has not been incorporated into any internationally risk assessment frameworks for the prediction of CAD [3–5, 10].

The hyperinsulinemic-euglycemic clamp technique serves as the standard for diagnosing insulin resistance, but its invasiveness, cost, and complexity make it unsuitable for epidemiological studies [11]. The Homeostasis Model Assessment of Insulin Resistance (HOMA-IR) is a commonly employed alternative, offering ease of use; however, this test cannot be used to diagnose people who are already undergoing insulin treatment [12, 13]. Additionally, HOMA-IR has another limitation, as laboratories do not routinely measure circulating insulin concentrations [14, 15].

In light of the drawbacks of direct measurement of insulin, numerous surrogate markers, based on glucose and lipid profiles as well as some anthropometric features, have emerged. These surrogate markers do not necessitate the measurement of serum insulin levels, and they have an even better correlation with the hyperinsulinemic-euglycemic clamp method compared to HOMA-IR [16–18]. The ratio of triglycerides to high-density lipoprotein cholesterol (TG/HDL-C), triglyceride-glucose index (TyG index), TyG-index with body mass index (TyG-BMI), TyG index with waist circumference (TyG-WC), and metabolic score for insulin resistance (METS-IR), are the most common of these less complicated and practical markers [19, 20]. Although prior studies have shown associations between these indices and CAD, there is no specific threshold for utilizing these indices, and it remains uncertain which one of them better predicts CAD [21–23].

Determining the most reliable predictor among these comparable indices poses a significant challenge in clinical environments, where they can aid in screening and preventive measures to reduce CAD. In this regard, in addition to the conventional statistical methods, we have decided to employ embedded feature selection techniques, which involve the fusion of machine learning algorithms with the process of selecting features [22, 23]. The main advantage of these machine learning algorithms over traditional statistical methods is their reduced emphasis on hypothesis-driven inference [24, 25]. Instead, they prioritize predictive accuracy and can algorithmically derive covariate interactions [24, 26]. These characteristics enable us to evaluate the impact of each feature on CAD prediction comprehensively.

To determine which of these indices best predict CAD occurrence, we first investigated the association between different surrogate markers of insulin resistance and CAD in a 10-year prospective cohort study. Then, we evaluated the optimal cut-off points for these surrogate markers as CAD prediction tools. The ultimate objective was to develop embedded feature selection machine learning algorithms for CAD prediction and to compare the unique impacts of insulin resistance markers on CAD prediction.

## Materials and methods

### Study population

Data for this cohort study were derived from the Yazd Healthy Heart Project (YHHP), an epidemiological study investigating cardiovascular and metabolic illnesses in a population-based setting. In summary, a total of 2000 Iranian adults (1000 men and 1000 women) between the ages of 20 and 74 were selected using a cluster random sampling technique. The participants were recruited from the urban population of Yazd city during the period of 2005–2006 [27].

### Inclusion and exclusion criteria

**Fig. 1:**
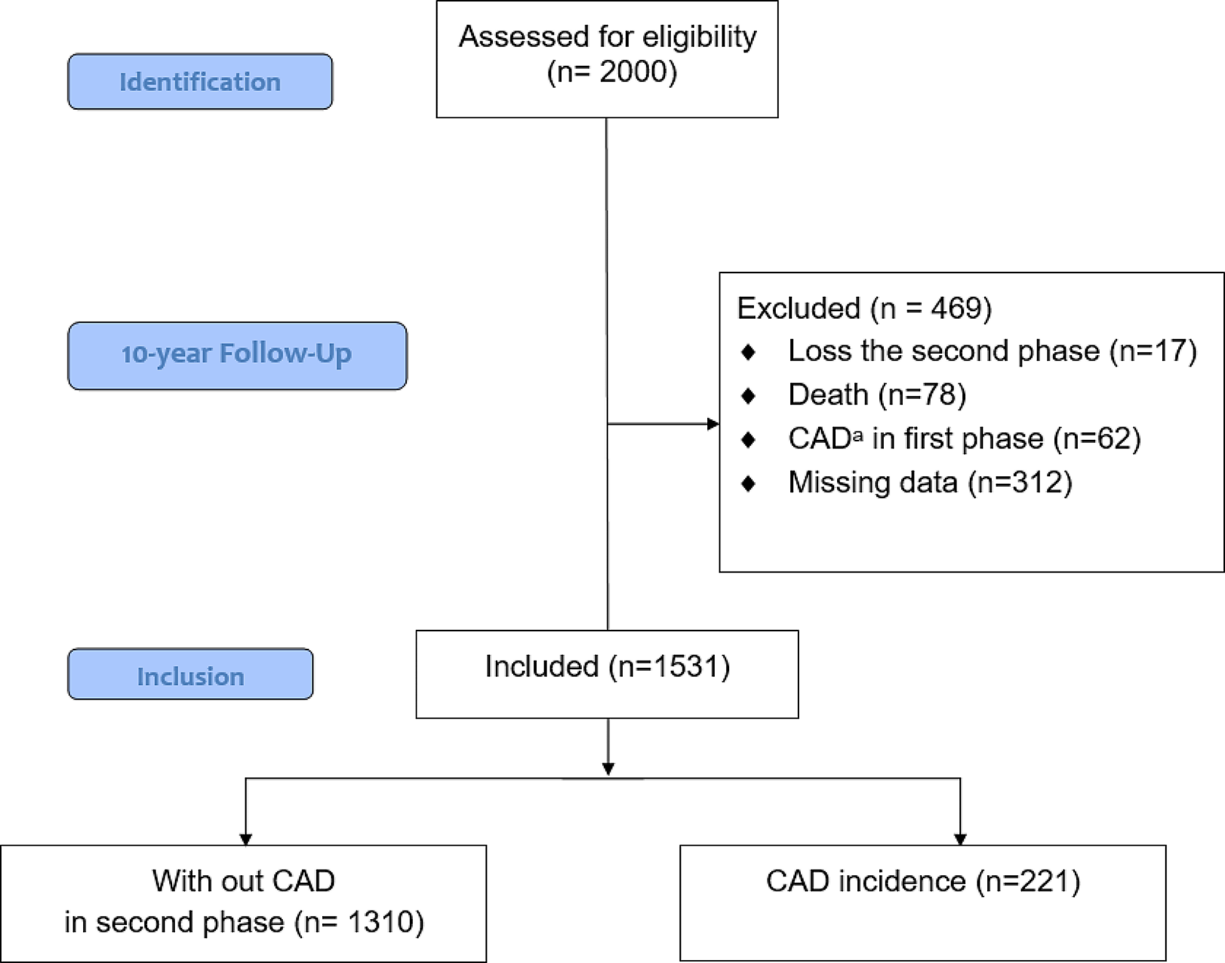
Flow diagram of participants attending the 10-year follow-up study. ^a^Coronary Artery Disease

 From the 2000 participants, 17 were omitted from the study due to loss during the second phase; from the 1983 individuals participating in the baseline examination, 62 were excluded due to diagnosis of CAD at baseline, 78 due to death during the study, and 312 due to missing data. The remaining 1531 participants (791 men, mean age 48.6 ± 14.7 years) were included in the present study (Fig. 1).

### Biochemical analyses

Lab analyses were conducted following an overnight fasting. Glucose and triglyceride (TG) levels were measured following centrifugation using kits obtained from Pars Azmoon Inc.(Tehran, Iran). The lipid profiles, including total cholesterol, low-density lipoprotein (LDL), and high-density lipoprotein (HDL), were examined using Bionic kits manufactured by Bionic Company (Tehran, Iran). The tests were conducted utilizing a biochemical autoanalyzer (BT 3000, Italy). The key exposure variables of interest were calculated using the following equations [18]:

$$\varvec{TyG-}\varvec{index}= \text{ln} \left(\frac{Tg \left(\frac{mg}{dl}\right)\times FBS\left(\frac{mg}{dl}\right)}{2}\right)$$

$$\varvec{TyG-}\varvec{BMI}=TyG-index \times BMI\left(\frac{Kg}{{m}^{2}}\right)$$

$$\begin{aligned} \varvec{TyG-}\varvec{WC}=&\,TyG-index \\& \times \text{Waist circumferance} \left(cm\right) \end{aligned}$$

$$\begin{aligned} \varvec{METS-}\varvec{IR}=&\,\text{ln}\Bigg(\bigg(2\times FBS\bigg(\frac{mg}{dl}\bigg)\bigg) \\& +\bigg(Tg\big(\frac{mg}{dl}\big)\times \frac{BMI\big(\frac{kg}{{m}^{2}}\big)}{\text{ln}\big(HDL\big(\frac{mg}{dl})}\bigg)\Bigg) \end{aligned}$$

$$\varvec{TG-}\varvec{HDL}\, \varvec{ration}=\frac{TG\left(\frac{mg}{dl}\right)}{HDL\left(\frac{mg}{dl}\right)}$$

### Anthropometric features

The participants’ heights were measured with a stadiometer attached to a smooth wall with no dents or irregularities. They stood barefoot, with their heels, hips, shoulders, and heads touching the wall and fixed horizontally. The heights were measured with a 0.5 centimeter margin of error. Participants were weighed with minimal clothing on a digital scale (Seca, Germany). The participants’ weight was measured with precision to the nearest 0.1 kg in both phases. The circumferences of the waist and hips were measured using a non-stretchable tape at the superior border of the iliac crest and the widest part of the buttock, respectively.

### Blood pressure measurements

The participants’ right arm blood pressure was measured by an Omron M6 comfort digital automatic blood pressure monitor in a sitting position. Nursing staff measured blood pressure twice, with a five-minutes interval between measurements.

### Physical activity, family history of premature CAD, smoking, and education

Trained interviewers utilized questionnaires to gather demographic information, physical activity, smoking habits, family history of early premature CAD, and angina pectoris. The assessment of physical activity was conducted using the International Physical. Activity Questionnaire (IPAQ) [28]. As part of this survey, the participants were questioned about the duration and number of days of their walking, engagement in moderate intensity exercise, and strenuous activity. Based on these inquiries, the number of MET-hours per week was computed, which is equivalent to 1 kcal/kg/hr [29]. Using this metric, the participants were categorized into low-, moderate-, and high-activity groups. Based on current smoking habits, the participants were categorized into two groups: smokers and nonsmokers. Family history of premature CAD was defined by the occurrence of CAD in a mother or sister before the age of 55, or in a father or brother before the age of 45.

### Outcome definition

CAD events were identified based on medical records documenting occurrences of fatal or nonfatal CAD, myocardial infarction, coronary artery bypass graft, positive exercise tests, positive cardiac enzymes, and positive percutaneous coronary angiography. In addition, all participants completed the Rose angina questionnaire (RAQ) [30], a validated tool for assessing new angina. The participants also had electrocardiograms (ECG), which were reviewed by both a general practitioner and a trained nurse. If any discrepancies arose, a cardiologist confirmed the findings. In addition to medical records, CAD was classified as having positive RAQ and findings of ischemia in the ECG.

### Statistical analysis

SPSS version 27.0 (IBM Corp., Armonk, NY, USA), Python 3, and R version 4.2.2 (www.R-project.org) were used for statistical analysis. Continuous variables were described as mean ± standard deviation (SD) and compared by ANOVA. Chi-square tests were used to compare categorical variables as numbers (percentages).

We employed multivariable Cox proportional hazard models to assess the association between quartiles of these indices and the CAD incidence. We employed two multivariable models for adjustment. Model 1 was adjusted for age and sex, whereas model 2 was adjusted for model 1 plus systolic and diastolic blood pressure, total cholesterol, LDL, HDL, BMI, waist to hip ratio, family history of premature CAD, physical activity, and smoking. If any of these factors were included in exposure variables (surrogate insulin resistance indices), we excluded them from the adjustment process. For instance, when analyzing TG/HDL ratio, we did not incorporate HDL into the statistical model.

We employed the receiver operative characteristic (ROC) curve to compare the predictive performance of all indices relative to one another. Then, we assessed the optimal cutoff points of surrogate insulin resistance indices with maximum sensitivity and specificity simultaneously, maximum, negative and positive diagnostic ratio, as well as maximum Youden index for predicting CAD using “OptimalCutpoints” R package [31]. In addition, we categorized these thresholds according to gender.

In order to choose the best surrogate insulin resistance marker for predicting CAD, we combined integrative methods with an ensemble of different embedded feature selection methods based on machine learning [23]. For integrative part of our approach, we selected age, sex, systolic blood pressure (SBP), diastolic blood pressure (DBP), LDL, total cholesterol, smoking, family history of premature CAD, and diabetes as our reference variables for comparing our surrogate measures of insulin resistance. For the embedded feature selection part, at first, we used random forest feature selection, which is a non-linear algorithm which can consider multiple interactions and evaluate variables by determining how much each feature can reduce impurities (Mean Decrease in Impurity [MDI]) [32]. For the second approach, we employed the Boruta algorithm, which shuffles the values of each feature and creates shadow features, which represent noise or irrelevant features, then trains a random forest model on original features and shadow features and compares their importance in multiple iterations. If a feature is more important than its shadow, it will be selected [33]. As a third approach, we used least absolute shrinkage and selection operator(LASSO), a regularization technique based on linear regression which drives the coefficients of less important features to zero and selects non-zero coefficient variables [34]. We set the alpha (threshold of significance) to 0.05 for this algorithm. Finally, we used ceteris paribus profile of the random forest model [35, 36]. The ceteris paribus profile can graphically depict the effect of altering specific variables on the predictive performance of the model while keeping all other elements unchanged.

## Results

### Association of surrogate insulin resistance indices with CAD

Table 1 presents the baseline characteristics of participants according to quartiles of surrogate insulin resistance indices. Age, blood pressure, low education, total cholesterol levels, and LDL showed a significant difference between quartiles for all markers. Table 2 reports the association between different surrogate markers of insulin resistance and CAD incidence. In model 1, after age and sex adjustments, the highest values among all indices in the fourth quartile were significantly and positively associated with CAD. Nevertheless, following adjustment for multiple variables in model 2, only the TyG-index was significantly associated with CAD (hazard ratio [HR]: 2.54, Confidence Interval [CI]: 1.34–4.81, P value = 0.007, P trend = 0.02). Only the TG/HDL ratio in men (HR: 1.95, CI: 1.01–3.77, P value = 0.04, P trend = 0.07) and TyG-index in women (HR: 4.76, CI: 1.36–16.66, P value = 0.01, P trend = 0.004) were associated with CAD after final adjustment (Table 3).

**Table 1:** Baseline characteristics of the participants according to quartiles of different surrogate markers of insulin resistance

	Q1	Q2	Q3	Q4	*P* value
**TyG-index**					
Age (years)	41.1 ± 15.7	48.1 ± 14.7	50.2 ± 13.7	53.8 ± 11.8	< 0.001
Male (%)	168 (47.5)	219 (57)	194 (50.5)	197 (50.9)	0.06
Systolic blood pressure (mm Hg)	120.9 ± 13.9	127.4 ± 14.8	129.7 ± 14.6	134 ± 15.7	< 0.001
Diastolic blood pressure (mm Hg)	79.2 ± 7.9	82.1 ± 8.2	83.8 ± 9	84.9 ± 8.6	< 0.001
Low density lipoprotein cholesterol (mg/dl)	95 ± 33	110.1 ± 35.4	116.5 ± 33	114.5 ± 41.6	< 0.001
Total cholesterol (mg/dl)	171.7 ± 38.9	192.4 ± 39.2	207.1 ± 37.4	223.7 ± 47.8	< 0.001
Family history of premature CAD (%)	39 (11.1)	48 (12.7)	67 (18)	65 (17.1)	0.02
**Physical activity**					0.005
Low (%)	139 (61)	162 (63)	186 (70.5)	207 (75.3)	
Moderate (%)	78 (34.2)	81 (31.5)	68 (25.8)	53 (19.3)	
High (%)	11 (4.8)	14 (5.4)	10 (3.8)	15 (5.5)	
**Education**					< 0.001
Primary (%)	156 (46.8)	210 (55.9)	237 (62.7)	273 (71.7)	
High school (%)	143 (42.9)	115 (30.6)	102 (27)	86 (22.6)	
Academic (%)	34 (10.2)	51 (13.6)	39 (10.3)	22 (5.8)	
Smoking (%)	55 (15.5)	79 (20.6)	71 (18.5)	67 (17.3)	0.34
**TyG-BMI**					
Age (years)	42.8 ± 17.3	49.6 ± 14.4	49.6 ± 13.1	51.7 ± 12.4	< 0.001
Male (%)	222 (61.2)	210 (56.8)	208 (53.5)	147 (36.7)	< 0.001
Systolic blood pressure (mm Hg)	121.4 ± 14.3	128.3 ± 15.5	130.4 ± 15.1	132.3 ± 14.8	< 0.001
Diastolic blood pressure (mm Hg)	79.2 ± 8.3	82.2 ± 8.8	83.9 ± 8.7	84.8 ± 8.3	< 0.001
Low density lipoprotein cholesterol (mg/dl)	98.3 ± 36.3	108.4 ± 34.4	112.3 ± 33.4	117.2 ± 39.9	< 0.001
Total cholesterol (mg/dl)	177.7 ± 42.3	194.1 ± 39.6	204.2 ± 39.8	218.8 ± 48	< 0.001
Family history of premature CAD (%)	33 (9.2)	62 (17.2)	58 (15.2)	68 (17.3)	0.005
**Physical activity**					0.57
Low (%)	159 (64.1)	178 (68.7)	188 (68.1)	179 (71)	
Moderate (%)	78 (31.5)	70 (27)	71 (25.7)	62 (24.6)	
High (%)	11 (4.4)	11 (4.2)	17 (6.2)	11 (4.4)	
**Education**					< 0.001
Primary (%)	173 (51.2)	197 (54.1)	241 (62.9)	275 (69.3)	
High school (%)	125 (37)	120 (33)	108 (28.2)	97 (24.4)	
Academic (%)	40 (11.8)	47 (12.9)	34 (8.9)	25 (6.3)	
Smoking (%)	82 (22.6)	79 (21.4)	68 (17.5)	47 (11.7)	< 0.001
**TyG-WC**					
Age (years)	40.5 ± 16.3	47.6 ± 14.1	51.1 ± 13.2	54.1 ± 11.7	< 0.001
Male (%)	193 (53.8)	188 (50.1)	198 (50.8)	212 (52.1)	0.77
Systolic blood pressure (mm Hg)	119.3 ± 13	127.1 ± 14.5	130.4 ± 15	135.1 ± 15	< 0.001
Diastolic blood pressure (mm Hg)	78.4 ± 7.5	82.2 ± 8.7	83.6 ± 8.5	85.7 ± 8.6	< 0.001
Low density lipoprotein cholesterol (mg/dl)	96.9 ± 34.2	111.3 ± 35.5	110 ± 35.6	117.9 ± 38.2	< 0.001
Total cholesterol (mg/dl)	176 ± 40.2	196.5 ± 40.9	200.8 ± 40.2	220.9 ± 46.9	< 0.001
Family history of premature CAD (%)	37 (10.4)	64 (17.4)	62 (16.3)	58 (14.5)	0.04
**Physical activity**					0.04
Low (%)	143 (60.9)	161 (64.9)	191 (71)	212 (73.4)	
Moderate (%)	80 (34)	76 (30.6)	66 (24.5)	62 (21.5)	
High (%)	12 (5.1)	11 (4.4)	12 (4.5)	15 (5.2)	
**Education**					< 0.001
Primary (%)	155 (45.9)	201 (54.9)	242 (63)	292 (72.6)	
High school (%)	144 (42.6)	114 (31.1)	108 (28.1)	87 (21.6)	
Academic (%)	39 (11.5)	51 (13.9)	34 (8.9)	23 (5.7)	
Smoking (%)	64 (17.8)	75 (20)	61 (15.6)	78 (19.2)	0.42
**TG/HDL ratio**					
Age (years)	44.9 ± 16.4	47.7 ± 14.9	50.9 ± 13.8	50.5 ± 13	< 0.001
Male (%)	166 (45.1)	199 (52.5)	184 (50.7)	242 (57.5)	0.01
Systolic blood pressure (mm Hg)	123.3 ± 15.5	127 ± 14.3	130.3 ± 15.4	131.9 ± 15.3	< 0.001
Diastolic blood pressure (mm Hg)	80.3 ± 8.2	82.2 ± 8	83.6 ± 9.2	84.2 ± 9	< 0.001
Low density lipoprotein cholesterol (mg/dl)	98.5 ± 34.9	109.7 ± 33.8	115.5 ± 35.4	112.9 ± 40	< 0.001
Total cholesterol (mg/dl)	181.1 ± 42.1	192 ± 39.1	203.9 ± 42.6	217.6 ± 47.2	< 0.001
Family history of premature CAD (%)	43 (11.8)	51 (13.6)	58 (16.6)	69 (16.6)	0.18
**Physical activity**					0.03
Low (%)	135 (62.2)	174 (64.9)	176 (69.3)	222 (73.5)	
Moderate (%)	70 (32.3)	79 (29.5)	72 (28.3)	63 (20.8)	
High (%)	12 (5.5)	15 (5.6)	6 (2.4)	17 (5.6)	
**Education**					0.01
Primary (%)	184 (51.8)	215 (59.1)	228 (63.3)	263 (64)	
High school (%)	136 (38.3)	107 (29.4)	100 (27.8)	110 (26.8)	
Academic (%)	35 (9.9)	42 (11.5)	32 (8.9)	38 (9.2)	
Smoking (%)	47 (12.8)	67 (17.7)	72 (19.8)	92 (21.9)	0.01
**METS-IR**					
Age (years)	43.8 ± 17.4	49.6 ± 13.9	49.9 ± 13.9	50.5 ± 12.5	< 0.001
Male (%)	213 (58.5)	216 (57)	195 (49.7)	167 (42.2)	< 0.001
Systolic blood pressure (mm Hg)	121.7 ± 14.4	128.7 ± 15.1	129.7 ± 15.2	132.3 ± 3	< 0.001
Diastolic blood pressure (mm Hg)	79.4 ± 8	82.4 ± 8.3	83.5 ± 8.8	84.9 ± 8.9	< 0.001
Low density lipoprotein cholesterol (mg/dl)	98.2 ± 36.1	110.2 ± 34.5	111.1 ± 33.3	116.9 ± 40	< 0.001
Total cholesterol (mg/dl)	181.9 ± 43.9	198.1 ± 41.4	200.8 ± 41.1	214.7 ± 47.7	< 0.001
Family history of premature CAD (%)	37 (10.3)	64 (17.3)	50 (13)	70 (18)	0.57
**Physical activity**					0.7
Low (%)	156 (63.9)	180 (67.2)	189 (70)	182 (70.3)	
Moderate (%)	77 (31.6)	73 (27.2)	69 (25.6)	12 (4.6)	
High (%)	11 (4.5)	15 (5.6)	12 (4.4)	65 (25.1)	
**Education**					< 0.001
Primary (%)	175 (51)	207 (56.1)	245 (63.3)	263 (67.3)	
High school (%)	124 (36.2)	121 (32.8)	108 (27.9)	100 (25.6)	
Academic (%)	44 (12.8)	41 (11.1)	34 (8.8)	28 (7.2)	
Smoking (%)	70 (19.2)	81 (21.4)	63 (16.1)	64 (16.2)	0.16

**Table 2:** Risk of CAD according to quartiles of Surrogate markers of insulin resistance

Surrogate markers	Crude model		Model 1		Model 2	
	HR (95% CI)	*P* value	HR (95% CI)	*P* value	HR (95% CI)	*P* value
**TG/HDL-c**						
Q1	Ref		Ref		Ref	
Q2	1.16 (0.74–1.82)	0.52	1.16 (0.74–1.81)	0.53	1.38 (0.78–2.43)	0.27
Q3	1.65 (1.08–2.51)	0.02	1.38 (0.91–2.11)	0.13	1.48 (0.85–2.56)	0.17
Q4	1.96 (1.32–2.92)	0.001	1.75 (1.17–2.60)	0.01	1.66 (0.96–2.88)	0.07
P for trend	< 0.001		0.002		0.08	
**TyG-index**						
Q1	Ref		Ref		Ref	
Q2	1.95 (1.17–3.23)	0.01	1.51 (0.91–2.50)	0.11	1.81 (0.98–3.33)	0.06
Q3	2.20 (1.35–3.59)	0.002	1.67 (1.02–2.72)	0.04	1.69 (0.91–3.16)	0.1
Q4	4.03 (2.56–6.34)	< 0.001	2.60 (1.65–4.10)	< 0.001	2.54 (1.34–4.81)	0.004
P for trend	< 0.001		< 0.001		0.007	
**TyG-BMI**						
Q1	Ref		Ref		Ref	
Q2	2.04 (1.31–3.17)	0.002	1.86 (1.19–2.89)	0.01	1.63 (0.98–2.74)	0.06
Q3	1.80 (1.15–2.81)	0.01	1.58 (1.01–2.47)	0.05	1.07 (0.62–1.83)	0.81
Q4	2.31 (1.50–3.55)	< 0.001	2.16 (1.39–3.34)	0.001	1.29 (0.74–2.25)	0.37
P for trend	< 0.001		0.003		0.97	
**TyG-WC**						
Q1	Ref		Ref		Ref	
Q2	1.31 (0.80–2.12)	0.28	1.07 (0.66–1.74)	0.79	0.95 (0.52–1.76)	0.88
Q3	2.07 (1.34–3.22)	0.001	1.44 (0.93–2.24)	0.10	1.55 (0.86–2.78)	0.14
Q4	2.69 (1.77–4.09)	< 0.001	1.68 (1.10–2.56)	0.02	1.59 (0.82–3.08)	0.17
P for trend	< 0.001		0.003		0.06	
**METS-IR**						
Q1	Ref		Ref		Ref	
Q2	1.44 (0.94–2.20)	0.10	1.35 (0.88–2.07)	0.17	1.22 (0.75-2.00)	0.42
Q3	1.55 (1.03–2.36)	0.04	1.40 (0.92–2.12)	0.11	1.24 (0.76–2.03)	0.38
Q4	1.71 (1.14–2.57)	0.01	1.65 (1.10–2.48)	0.02	1.08 (0.65–1.79)	0.75
P for trend	0.01		0.02		0.89	

Model 1: adjusted for age and sex, Model 2: model 1 plus systolic and diastolic blood pressure, total cholesterol, LDL, HDL, BMI, waist to hip ratio, family history of premature CAD, physical activity, and smoking*

*If any of these factors were included in exposure variables (surrogate insulin resistance indices), we excluded them from the adjustment process

**Table 3:** Risk of CAD according to quartiles of Surrogate markers of insulin resistance stratified by gender

Surrogate markers	MEN						WOMEN					
	Crude model		Model 1		Model 2		Crude model		Model 1		Model 2	
	HR (95% CI)	*P* value	HR (95% CI)	*P* value	HR (95% CI)	*P* value	HR (95% CI)	*P* value	HR (95% CI)	*P* value	HR (95% CI)	*P* value
**TG/HDL-c**												
Q1	Ref		Ref		Ref		Ref		Ref		Ref	
Q2	1.36 (0.78–2.36)	0.28	1.50 (0.86–2.61)	0.15	1.6 (0.80–3.18)	0.18	1.06 (0.50–2.22)	0.88	0.83 (0.40–1.75)	0.63	1.1 (0.40–3.04)	0.86
Q3	1.39 (0.80–2.41)	0.24	1.39 (0.80–2.61)	0.24	1.29 (0.63–2.62)	0.48	2.18 (1.14–4.16)	0.02	1.44 (0.75–2.77)	0.27	1.42 (0.55–3.63)	0.47
Q4	2.26 (1.36–3.77)	0.002	2.26 (1.35–3.77)	0.002	1.95 (1.01–3.77)	0.04	1.92 (1.01–3.68)	0.048	1.33 (0.69–2.54)	0.39	1.14 (0.43–3.03)	0.79
P for trend	0.001		0.003		0.07		0.01		0.16		0.74	
**TyG-index**												
Q1	Ref		Ref		Ref		Ref		Ref		Ref	
Q2	1.21 (0.69–2.13)	0.51	1.08 (0.61–1.92)	0.78	2.09 (1.02–4.26)	0 0.04	2.03 (0.81–5.09)	0.13	1.23 (0.49–3.12)	0.66	1.4 (0.39–4.94)	0.60
Q3	1.31 (0.76–2.26)	0.33	1.22 (0.71–2.11)	0.47	1.48 (0.70–3.14)	0.31	3.58 (1.55–8.29)	0.003	1.95 (0.83–4.57)	0.12	2.27 (0.70–7.37)	0.17
Q4	2.50 (1.54–4.06)	< 0.001	2.00 (1.22–3.25)	0.005	1.93 (0.93–4.02)	0.08	6.14 (2.77–13.63)	< 0.001	2.97 (1.32–6.71)	0.01	4.76 (1.36–16.66)	0.01
P for trend	< 0.001		0.002		0.23		< 0.001		< 0.001		0.004	
**TyG-BMI**												
Q1	Ref		Ref		Ref		Ref		Ref		Ref	
Q2	1.65 (0.96–2.86)	0.07	1.57 (0.90–2.71)	0.11	1.86 (1.00-3.45)	0.05	1.58 (0.79–3.16)	0.19	1.32 (0.66–2.64)	0.43	0.96 (0.33–2.79)	0.94
Q3	1.63 (0.94–2.82)	0.08	1.48 (0.86–2.57)	0.16	0.99 (0.52–1.91)	0.98	2.17 (1.12–4.21)	0.02	1.80 (0.93–3.50)	0.08	1.11 (0.39–3.15)	0.76
Q4	2.2 (1.31–3.69)	0.003	1.89 (1.13–3.18)	0.02	1.45 (0.69–3.05)	0.32	2.19 (1.14–4.22)	0.02	1.60 (0.83–3.08)	0.16	0.79 (0.27–2.27)	0.66
P for trend	0.004		0.03		0.99		0.01		0.12		0.92	
**TyG-WC**												
Q1	Ref		Ref		Ref		Ref		Ref		Ref	
Q2	1.48 (0.82–2.66)	0.16	1.35 (0.75–2.42)	0.32	1.34 (0.63–2.84)	0.44	1.40 (0.64–3.06)	0.39	0.95 (0.44–2.09)	0.91	0.36 (0.11–1.15)	0.08
Q3	1.93 (1.11–3.38)	0.02	1.43 (0.82–2.51)	0.21	1.47 (0.69–3.13)	0.31	2.39 (1.19–4.81)	0.01	1.31 (0.65–2.65)	0.46	1.17 (0.43–3.17)	0.76
Q4	2.74 (1.62–4.62)	< 0.001	1.91 (1.13–3.24)	0.02	1.44 (0.75–4.06)	0.19	2.86 (1.45–5.62)	0.002	1.45 (0.73–2.88)	0.29	0.94 (0.28–3.08)	0.92
P for trend	< 0.001		0.01		0.2		< 0.001		0.15		0.34	
**METS-IR**												
Q1	Ref		Ref		Ref		Ref		Ref		Ref	
Q2	1.32 (0.76–2.29)	0.33	1.22 (0.70–2.13)	0.48	1.38 (0.75–2.56)	0.30	1.62 (0.87–3.02)	0.13	1.54 (0.83–2.88)	0.17	0.89 (0.38–2.11)	0.80
Q3	1.67 (0.98–2.83)	0.06	1.43 (0.84–2.43)	0.18	1.39 (0.74–2.62)	0.30	1.44 (0.76–2.73)	0.26	1.34 (0.71–2.53)	0.37	0.94 (0.40–2.17)	0.87
Q4	1.98 (1.18–3.30)	0.01	1.73 (1.04–2.90)	0.03	1.50 (0.79–2.85)	0.22	1.41 (0.75–2.63)	0.29	1.23 (0.66–2.31)	0.52	0.53 (0.22–1.29)	0.17
P for trend	0.005		0.025		0.27		0.43		0.74		0.13	

Model 1: adjusted for age and sex, Model 2: model 1 plus systolic and diastolic blood pressure, total cholesterol, LDL, HDL, BMI, waist to hip ratio, family history of premature CAD, physical activity, and smoking*

*If any of these factors were included in exposure variables (surrogate insulin resistance indices), we excluded them from the adjustment process

Table 4 presents the area under the ROC curve (AUC) and cut-off points for all indices used to predict CAD in men, women, and the total sample. The TyG-index demonstrated superior predictive performance in both the total sample and among women, with AUC values of 0.67 (0.63–0.70, P value 0.001) and 0.72 (0.66–0.77), respectively. However, the TyG-index and the TyG-WC revealed almost identical performance in men.

**Table 4:** Receiver operating characteristic curve and cut-off points of surrogate markers of insulin resistance for CAD prediction in men, women, and the total population
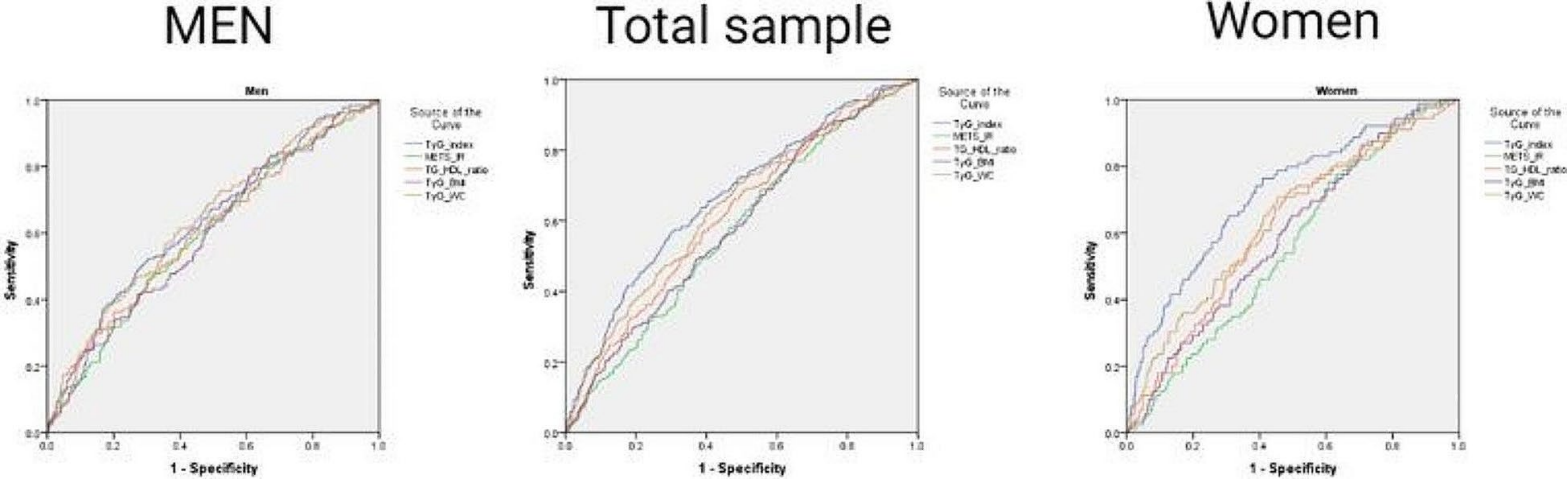

Surrogate marker	Performance		Cutoff points			
	AUC (95% CI)	*P* Value	Maximum sensitivity and specificity simultaneously	Maximum Youden index	Negative diagnostic ratio value	Positive diagnostic ratio value
**Total population**						
TyG-index	0.67 (0.63–0.7)	< 0.001	8.99	9.12	8.42	9.28
METS-IR	0.57 (0.53–0.61)	0.001	39.83	37.39	29.44	63.85
TG/HDL	0.61 (0.57–0.65)	< 0.001	3.13	2.79	1.47	5.79
TyG-WC	0.64 (0.60–0.68)	< 0.001	862.03	857.65	734.28	971.43
TyG-BMI	0.59 (0.55–0.63)	< 0.001	237.21	208.68	199.81	296.01
**Men**						
TyG-index	0.63 (0.58–0.68)	< 0.001	8.92	9.19	8.36	9.54
METS-IR	0.59 (0.54–0.65)	0.001	39.10	41.00	24.63	57.98
TG/HDL	0.60 (0.55–0.66)	< 0.001	3.24	3.15	1.11	5.82
TyG-WC	0.64 (0.59–0.69)	< 0.001	857.70	857.65	616.15	948.73
TyG-BMI	0.60 (0.55–0.66)	< 0.001	228.70	209.05	154.88	267.93
**Women**						
TyG-index	0.72 (0.66–0.77)	< 0.001	9.07	8.93	9.04	9.10
METS-IR	0.56 (0.50–0.61)	0.08	−	−	−	−
TG/HDL	0.61 (0.55–0.68)	< 0.001	3.02	2.67	1.43	7.82
TyG-WC	0.64 (0.58–0.70)	< 0.001	872.42	849.42	737.18	971.02
TyG-BMI	0.59 (0.53–0.65)	0.004	248.15	241.48	206.73	296.01

**Fig. 2:**
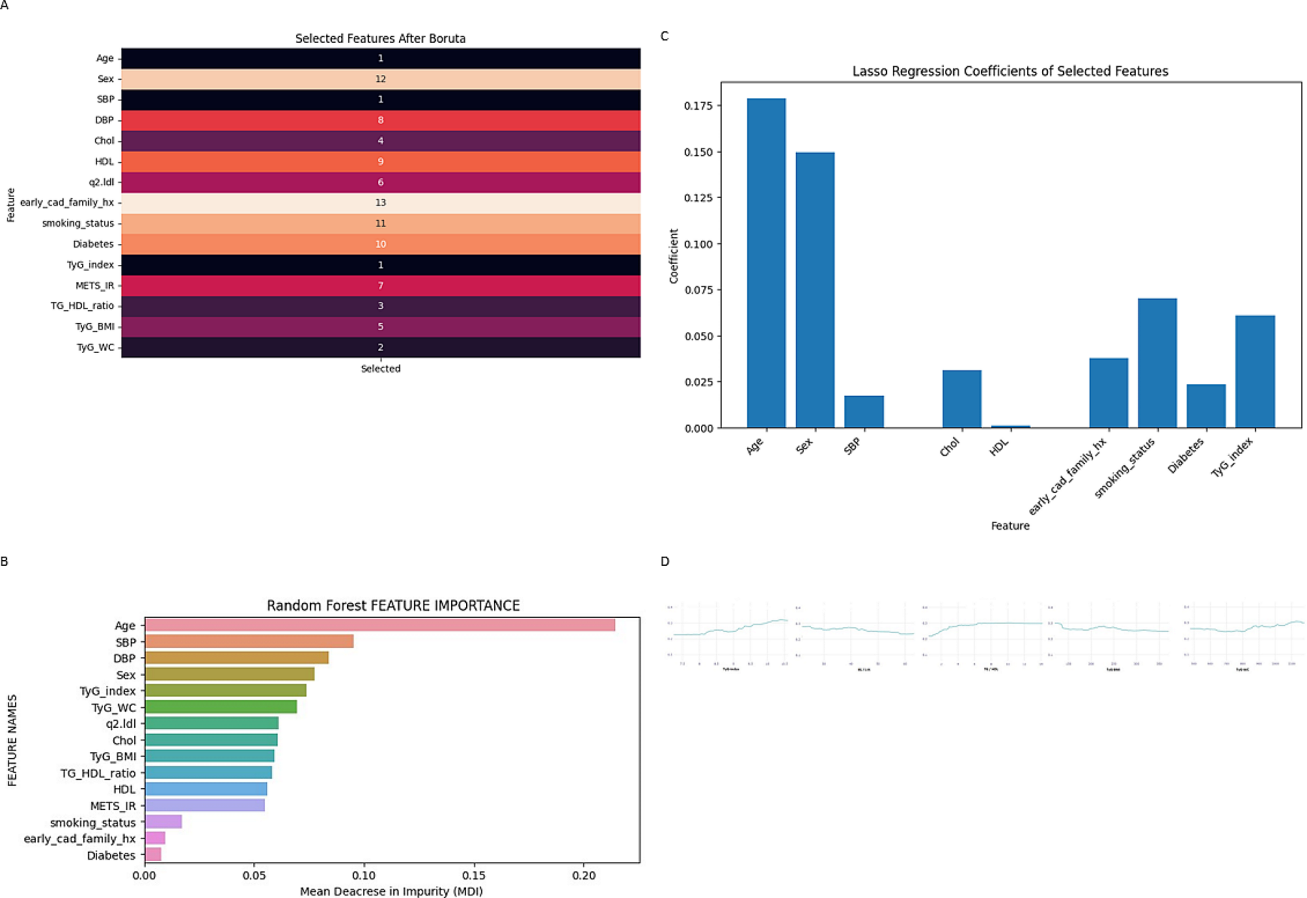
Ensemble of embedded feature selection methods. **A** This figure illustrates the Importance of variables based on their rank in the Boruta method, a lower rank indicates greater importance, while a higher rank indicates lesser importance. The variables highlighted in black are the most important ones. **B** The mean decrease in impurity (MDI) or Gini importance measures the extent to which every feature contributes to accurate predictions. A higher MDI value indicates that the variable is more important. **C** LASSO is a regularization approach based on linear regression. Regularization approaches penalize large coefficients because their presence can lead to overfitting. LASSO decreases coefficients of less significant features to zero and selects features that haven't been lowered to zero. A higher coefficient indicates greater importance. **D** The Ceteris paribus profile examines individual features while holding all other components of the model constant, in order to understand the particular impact of different features on predictions in machine learning models. A sharper incline on the diagram without a plateau or a downward slope with a higher constant indicate a better feature.

Figure 2 illustrates several feature selection methods and the ceteris paribus profile of a random forest model. Figure 2A indicates the feature selection process using the Boruta algorithm. According to this algorithm, age, SBP, and TyG-index were the most important variables for predicting CAD. The random forest model revealed that, following age, blood pressure, and sex, the TyG-index exhibited the greatest MDI, thus serving as the most effective surrogate measure of insulin resistance for predicting CAD (Fig. 2B).

Figure 2C depicts the LASSO technique, which is a penalized approach that discards redundant variables. The TyG-index was the only surrogate indicator of insulin resistance that was chosen by LASSO. The Ceteris paribus profile of a random forest model is shown in.

Figure 2D Compared to other indices, the TyG-index had a stronger positive slope without a clear plateau or decline.

## Discussion

Our research findings demonstrated that the TyG-index is the most effective surrogate marker of insulin resistance for predicting CAD and it has superior predictive capabilities in women. Not only did traditional statistical methods like Cox hazard regression and ROC analysis show that the TyG-index had a better HR and AUC for CAD compared to other surrogate indicators of insulin resistance, but also advanced feature selection techniques further validated these findings.

Surrogate insulin resistance markers encompass both blood glucose and dyslipidemia markers, serving as indirect indications of insulin resistance in the liver and adipose tissue [37]. Furthermore, some of these surrogate markers, including TyG-WC, TyG-BMI, and METS-IR, integrate obesity measures. This approach is grounded in the understanding that a direct relationship exists between insulin resistance and the majority of obesity indicators [38]. The advantage of these non-insulin dependent surrogate measures of insulin resistance, compared to the insulin-dependent competitors such as HOMA-IR, lies in their cost-effective and simplified acquisition technique, as well as their stronger association with the gold standard protocol for measuring insulin resistance [11–13]. Furthermore, research indicates that some of these indices may be more effective predictors of CAD than metabolic syndrome, which itself is a reflection of insulin resistance [39].

The findings from meta-analyses have shown a relationship between the TyG-index [40] and TG/HDL-C ratio [41] with CAD. Additionally, cohort studies have demonstrated the association of TyG-BMI and METS-IR with CAD [19, 42, 43], while only a cross-sectional study has highlighted a link between TyG-WC and CAD [19]. In the current study, TyG-BMI and METS-IR were not associated with CAD and were also found to be the least effective surrogate markers in the feature selection approaches. The potential explanation is in the fact that BMI fluctuations alone, as the sole anthropometric characteristic, fail to accurately indicate the risk of CAD when accompanied with insulin resistance-related traits [44, 45]. Although, in the present study, TyG-WC was the second most reliable indicator after TyG-index, we found no significant association with CAD.

To date, only four studies have directly compared surrogate markers of insulin resistance and their association with CAD within a single analytical framework [19–21, 46]. Among these, a case-control study highlighted the METS-IR index as more closely associated with CAD than both the TG/HDL and TyG-index, though this conclusion might be affected by Berkson’s bias due to the selection process, which targeted participants suspected of CAD and underwent coronary angiography [20]. Elsewhere, an analysis of cross sectional data from the National Health and Nutrition Examination Survey (NHANES) revealed a stronger correlation between the TyG-index and CAD, outperforming other indices, though the TyG-WC indicated a greater AUC [19]. However, the reliance on self-reported outcomes in NHANES study raises concerns about misclassification. Furthermore, research by Mahdavi-Roshan et al. in Iran, employing a case-control approach, indicated that the TyG-index was more closely associated with CAD risk than either the METS-IR or TyG-BMI [21]. Recently, Liu et al. in a prospective cohort of Chinese population evaluated visceral obesity indices and surrogate insulin resistance markers for predicting coronary heart disease [46]. They found that the Chinese visceral adiposity index (CVAI) is a more accurate predictor of coronary heart disease than surrogate markers of insulin resistance [46]. Although this index does have a correlation with insulin resistance and cardiometabolic disease, it was not initially designed for measuring insulin resistance. The initial development and validation of this index is based on measurements of Visceral adipose tissue (VAT) acquired through CT scan [47, 48]. Conversely, surrogate insulin resistance markers particularly formulated based on HOMA-IR and glucose clamp test [49, 50, 51, 52]. Furthermore, CVAI has been designed for people of Chinese ethnicity, which differs significantly from our community. For instance, in China, 34.3% of adults are overweight and 16.4% are obese [48]. In contrast, 63% of the Iranian population is overweight or obese, with 70.54% exhibiting abdominal obesity based on waist-to-hip ratio [53]. Although assessing these measures of visceral obesity is not within the scope of this study, it would be intriguing for future studies to determine which obesity indices are most effective in predicting CAD in the Persian population and whether they have a greater impact than indicators of insulin resistance. Overall, it is crucial to be cautious when interpreting these results because of inherent biases, differing findings among various studies, dependence on cross-sectional data, and reliance on traditional statistical methods. Accurately predicting intricate diseases such as CAD requires considering complex interactions among several parameters [23], a consideration that is overlooked in traditional techniques.

### Embedded feature selection

Embedded feature selection techniques are types of supervised learning dimension reduction techniques used to identify the optimal variables for predicting an outcome [53]. Not only do they enhance predictive models’ performance and cost-effectiveness [54], they can also help healthcare practitioners select the most appropriate variable from a set of variables that have similar information and overlap with each other for the goal of screening and preventing an outcome. Although there is no flawless integrated feature selection algorithm [55], we can combine these strategies to use their respective advantages and mitigate their limitations [56]. Nevertheless, it is important to acknowledge that the decision between using novel techniques such as machine learning and traditional statistical models in predictive analytics is not a clear-cut one. Traditional statistical models offer a transparent depiction of the data, often including a probabilistic framework, which enhances interpretability. These models highlight relevant variables and quantify the strength as well as significance of associations. Conversely, machine learning models tend to be more empirical, prioritizing predictive performance over interpretability. Previous research has indicated that the complementation of conventional statistical techniques and machine learning is the optimum strategy to guide to generalizable and significant findings [57]. This is why we employed both of these methods to achieve a more comprehensive interpretation of our data.

Ensemble of feature selection approaches in the current study indicated that the TyG-index is the best surrogate marker of insulin resistance for predicting CAD. Following that, the TyG-WC may have the greatest influence. Ceteris paribus profile of random forest model demonstrated that predictive capability of the TyG-index grew after 9 with a positive slope without any decline or flattening out, which was in accordance with the cutoff points of the ROC curve. The TyG-BMI and METS-IR curves displayed a consistently flat and negative slope, while the TG-HDL and TyG-WC curves showed various instances of plateauing or downhill, suggesting that they are not reliable indicators for predicting CAD.

The combination of all three embedded feature selection methods, along with the results of Cox hazard models and ROC curve analysis, demonstrated that the TyG-index is the most reliable surrogate insulin resistance index for predicting CAD. This consensus of findings of different methods demonstrates the stability and reproducibility of the result, thereby increasing confidence in the use of this index [57, 58] for CAD prediction.

### Strengths and limitations

This study is the first to evaluate and compare the most common surrogate measures of insulin resistance within a unified framework for the prediction of CAD. The prospective structure of our study, which has focused on the community, helps to limit the likelihood of reverse causation and recall bias. Unlike previous studies [19], we employed a consistent approach to define CAD by examining both paraclinical and symptomatic data. This enabled us to reduce the likelihood of misclassification.

This study also had some limitations. A few follow-up sessions would constrain our ability to assess and regulate voluntary health check-ups as well as lifestyle modifications that may have influenced our findings over the ten-year study period. Further, conducting a study on surrogate insulin resistance indices using a single baseline evaluation may cause our results to be influenced by differences within individuals over time. Above all, our study was conducted at a single center and included only individuals of the Iranian population. Thus, it is important to note that our findings may not be generalizable to populations in other countries.

## Conclusion

The findings of the present investigation indicate that the TyG-index is the most efficient surrogate insulin resistance index for predicting and preventing CAD. Given the ease of evaluating the TyG-index using routine biochemical tests, incorporating this tool into clinical screenings and including it in future CAD risk assessment scores can greatly enhance healthcare professionals’ ability to manage and lower the risk of CAD. Nevertheless, more research involving multiple centers and diverse ethnic groups is necessary to validate our results.

## Data Availability

The datasets used and/or analyzed during the current study are available from the corresponding author on reasonable request.

## Abbreviations

TyG-index: Triglyceride glucose index
TyG-BMI: Triglyceride glucose body mass index
TyG-WC: Triglyceride glucose waist circumference index
METS-IR: Metabolic score for insulin resistance
CVD: Cardiovascular disease
CAD: Coronary artery disease
DALY: Disability adjusted life years
HR: Hazard ratio
CI: confidence interval
HOMA-IR: Homeostatic Model Assessment for Insulin Resistance
YHHP: Yazd Healthy Heart Project
TG: Triglyceride
LDL: Low-density lipoprotein
HDL: High-density lipoprotein
FBS: Fasting blood sugar
IPAQ: International Physical Activity Questionnaire
RAQ: Rose angina questionnaire
SBP: Systolic blood pressure
DBP: Diastolic Blood pressure
SD: Standard deviation
BMI: Body mass index
ECG: Electrocardiogram
MDI: Mean decrease in impurity
LASSO: Least absolute shrinkage and selection operator
ROC: Receiver operating characteristic
AUC: Area under the curve
NHANES: National Health and Nutrition Examination Survey
